# p53/PUMA expression in human pulmonary fibroblasts mediates cell activation and migration in silicosis

**DOI:** 10.1038/srep16900

**Published:** 2015-11-18

**Authors:** Wei Wang, Haijun Liu, Xiaoniu Dai, Shencun Fang, Xingang Wang, Yingming Zhang, Honghong Yao, Xilong Zhang, Jie Chao

**Affiliations:** 1 Department of Respiratory Medicine, The First Affiliated Hospital of Nanjing Medical University, Nanjing, Jiangsu 210029, China; 2 Department of Physiology, Medical School of Southeast University, Nanjing, Jiangsu 210009, China; 3 Neurobiology Laboratory, New Drug Screening Centre, China Pharmaceutical University, Nanjing, Jiangsu 210009, China; 4 Nine Department of Respiratory Medicine, Nanjing Chest Hospital, Nanjing, Jiangsu 210029, China; 5 Department of Pharmacology, Medical School of Southeast University, Nanjing, Jiangsu 210009, China; 6 Key Laboratory of Developmental Genes and Human Disease, Southeast University, Nanjing, 210096, China

## Abstract

Phagocytosis of SiO_2_ into the lung causes an inflammatory cascade that results in fibroblast proliferation and migration, followed by fibrosis. Clinical evidence has indicated that the activation of alveolar macrophages by SiO_2_ produces rapid and sustained inflammation characterized by the generation of monocyte chemotactic protein 1, which, in turn, induces fibrosis. However, the details of events downstream of monocyte chemotactic protein 1 activity in pulmonary fibroblasts remain unclear. Here, to elucidate the role of p53 in fibrosis induced by silica, both the upstream molecular mechanisms and the functional effects on cell proliferation and migration were investigated. Experiments using primary cultured adult human pulmonary fibroblasts led to the following results: 1) SiO_2_ treatment resulted in a rapid and sustained increase in p53 and PUMA protein levels; 2) the MAPK and PI3K pathways were involved in the SiO_2_-induced alteration of p53 and PUMA expression; and 3) RNA interference targeting p53 and PUMA prevented the SiO_2_-induced increases in fibroblast activation and migration. Our study elucidated a link between SiO_2_-induced p53/PUMA expression in fibroblasts and cell migration, thereby providing novel insight into the potential use of p53/PUMA in the development of novel therapeutic strategies for silicosis treatment.

The inhalation of silicon dioxide particles causes pneumoconiosis, an untreatable pulmonary disease characterized by alveolar inflammation at the early stage and progressive lung fibrosis at the late stage. Considerable evidence has suggested that alveolar macrophages (AMOs) and pulmonary fibroblasts (PFBs) mediate pulmonary fibrosis, which results from chronic inflammation^1^,^2^. While the role of SiO_2_-induced chemokines and cytokines released from AMOs has received significant attention, the direct effect of SiO_2_ on functional protein production in PFBs has been studied less extensively.

Tumor protein 53 (p53) is a tumor suppressor that acts as a key component of the cellular emergency response mechanism by up-regulating growth arrest and apoptosis-related genes in response to a variety of extra- and intra-cellular stress signals^3^,^4^,^5^,^6^,^7^. Emerging evidence has suggested that p53 plays roles in not only cell differentiation, apoptosis, and cell cycle control but also modulation of cell migration and other functions^8^. Indeed, p53 has been reported to regulate the transcription of a number of genes. Among these are genes whose products mediate cell-matrix interactions and cell motility, such as HGF/scatter factor^9^, collagens IIa1 and Vla1, macrophage-stimulating protein (Msp), plasminogen activator inhibitor-1 (PAl-1)^10^, fibronectin^11^, VEGF^12^ and metalloproteinase-1^13^. p53-up-regulated modulator of apoptosis (PUMA) is a recently discovered Bcl-2 family member that is rapidly induced by p53 and that exerts strong pro-apoptotic effects. Recent studies have suggested that p53 and PUMA most likely participate in the fibrotic process in a synergistic manner^14^. However, the effects of p53 and PUMA on fibroblast regulation during silica-induced fibrosis remain unclear.

To elucidate the role of p53 in silica-induced fibrosis, we knocked down p53 expression and examined cellular behavior related to fibroblast-mediated contraction. Here, we demonstrated that p53 and PUMA are involved in the regulation of fibroblast contraction, growth and migration. These findings identified a new function of p53 in fibroblasts and suggested that p53 may be involved in multiple steps of the wound healing process.

## Results

### Effect of SiO_2_ on p53 expression in human pulmonary fibroblasts

**Figure 1:**
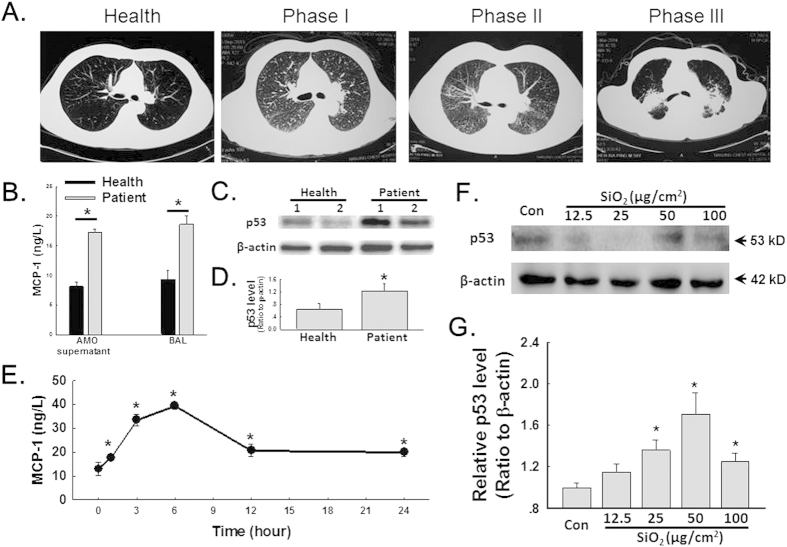
p53 expression increased in HPF-a either cultured in conditioned medium of AMOs derived from silicosis patients or directly exposed to SiO_2_. (**A**) Axial high-resolution CT sections of patients at different stages of silicosis. (**B**) ELISAs showing that the MCP-1 level in both the BAL fluid and the AMO culture medium from silicosis patients was significantly higher than that in samples from healthy donors. p < 0.05, n = 5. (**C**) Representative Western blot showing the effect of AMO-conditioned medium derived from patients or healthy donors on p53 expression in HPF-a. (**D**) Densitometric analyses of p53 expression from five healthy donors and five silicosis patients suggested that the p53 protein level in HPF-a cultured in medium from the patient group was significantly higher than the level in HPF-a cultured in medium from the healthy group. *p < 0.05. (**E**) ELISAs showing that SiO_2_ induced a rapid and sustained increase in MCP-1 expression in the supernatant of cultured AMOs from healthy donors. *p < 0.05 vs the 0 h group, n = 5. (**F**) Representative Western blot showing that SiO2 induced p53 expression in HPF-a in a dose-dependent manner. (**G**) Densitometric analyses of p53 (n = 5). *p < 0.05 vs the control group.

**Figure 2:**
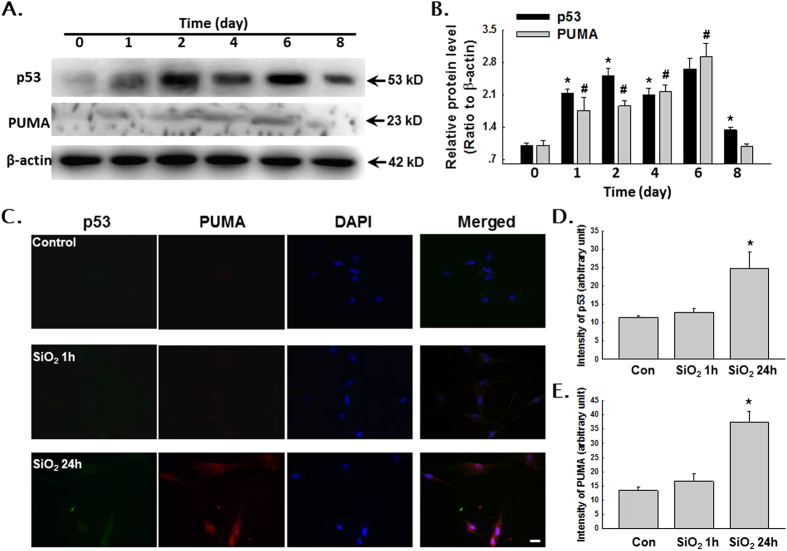
SiO_2_ induced both p53 and PUMA expression in HPF-a. (**A**) Representative Western blot showing that SiO_2_ induced both p53 and PUMA expression in HPF-a in a time-dependent manner. Scale bar = 10 μm. (**B**) Densitometric analyses of p53 and PUMA expression (n = 5). *p < 0.05 vs p53 expression on day 0; #p < 0.05 vs PUMA expression on day 0. (**C**) Representative immunocytochemical images showing that SiO_2_ increased the expression of p53 and PUMA in HPF-a. Densitometric analyses of p53 **(D)** and PUMA **(E**) expression (n = 5). *p < 0.05 vs the control group.

Accumulating evidence from our lab suggests that MCP-1 plays a critical role in SiO_2_-induced pulmonary fibrosis. Our current data suggested that MCP-1 expression is increased in not only the bronchoalveolar lavage (BAL) fluid from patients but also the supernatant of cultured AMOs (Fig. 1A,B). Moreover, the expression of p53 in adult human pulmonary fibroblasts (HPF-a) increased when the cells were treated with the supernatant of cultured AMOs from patients (Fig. 1C,D). Additionally, AMOs from healthy donors released MCP-1 after exposure to SiO_2_ in a time-dependent manner (Fig. 1E). Recent studies have suggested that p53 may serve as a pharmaceutical intervention against CCL2-mediated inflammatory responses^15^,^16^,^17^. To further understand the role of p53 in SiO_2_-induced fibrosis, we next examined the level of p53 in SiO_2_-treated HPF-a. As shown in Fig. 1F,G, SiO_2_ treatment of HPF-a resulted in the up-regulation of p53 expression in a dose-dependent manner. Because PUMA is known to be a downstream target gene of p53 that acts via p53-dependent and p53-independent pathways to promote apoptosis, we next measured the protein levels of both p53 and PUMA. As shown in Fig. 2A,B, the exposure of HPF-a to SiO_2_ induced rapid and sustained up-regulation of p53 and PUMA in a time-dependent manner. This result was confirmed by immunocytochemical staining (Fig. 2C–E).

### Effect of SiO_2_ on the phosphorylation of MAPKs and Akt

**Figure 3:**
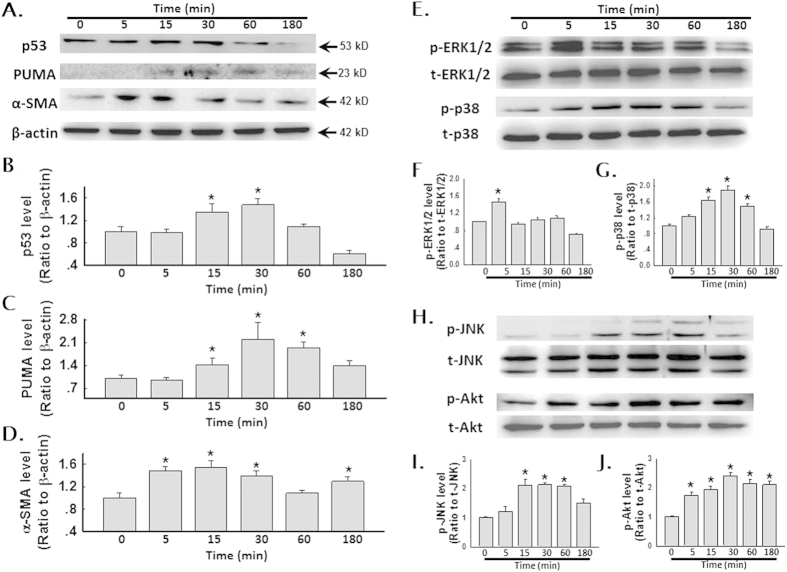
SiO_2_ induced the phosphorylation of MAPKs and PI3K/Akt in HPF-a. (**A**) Representative Western blot showing that SiO_2_ induced a rapid increase in p53, PUMA and α-SMA expression in HPF-a. Densitometric analyses of p53 **(B)**, PUMA **(C)** and α-SMA **(D)** expression from five separate experiments. *p < 0.05 vs the 0 min group. (**E**) Representative Western blot showing that SiO_2_ induced the rapid and transient phosphorylation of ERK and p38 in HPF-a. Densitometric analyses of p-ERK **(F)** and p-p38 **(G)** expression from five separate experiments. *p < 0.05 vs the 0 min group. (**H**) Representative Western blot showing that SiO_2_ induced the rapid phosphorylation of JNK and Akt in HPF-a. Densitometric analyses of p-JNK **(I)** and p-Akt **(J)** expression from five separate experiments. *p < 0.05 vs the 0 min group.

To further understand the molecular mechanism underlying SiO_2_-induced fibrosis, we investigated the potential association between kinase activation and p53 and PUMA expression. Thus, we measured the protein levels of both p53 and PUMA in HPF-a within 3 h of exposure to SiO_2_. The protein level of α-SMA (a marker of fibroblast activation) was also examined. As shown in Fig. 3A–D, the levels of all proteins investigated peaked after approximately 15–60 min of SiO_2_ exposure and then tapered off. Next, we measured the phosphorylation of MAPKs and PI3K/Akt within 3 h of exposure to SiO_2_. As shown in Fig. 3E,F, Erk phosphorylation increased and then diminished within 5 min of exposure to SiO_2_. After 15 min of exposure to SiO_2_, p38 was phosphorylated, peaking after approximately 30–60 min and then tapering off (Fig. 3E,F). JNK also demonstrated a burst of activation from 15 to 60 min after exposure to SiO_2_ (Fig. 3H,I). In contrast, Akt displayed rapid and sustained phosphorylation after exposure to SiO_2_.

### Effect of the pharmacological inhibition of MAPKs or Akt on p53 and PUMA induction after exposure to SiO_2_

**Figure 4:**
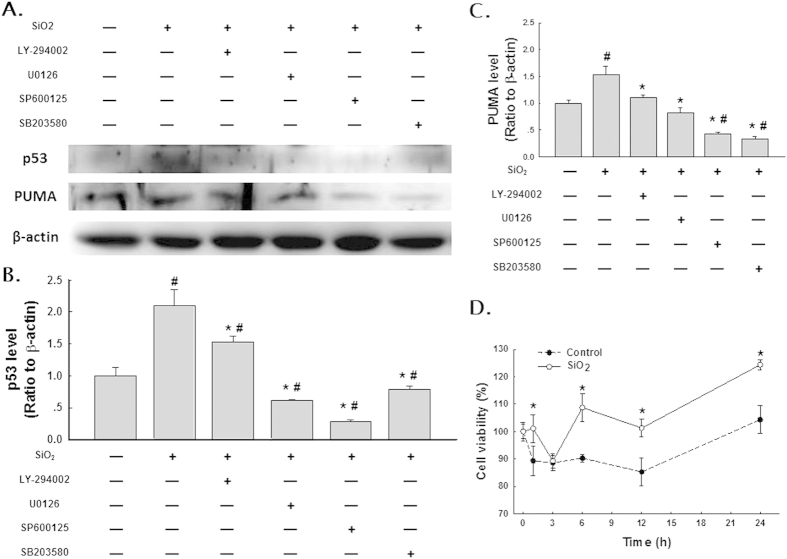
SiO_2_ induced p53 and PUMA expression in HPF-a via the MAPK and PI3K/Akt pathways. (**A**) Representative Western blot showing that p53 and PUMA expression induced by SiO_2_ was attenuated by pretreating HPF-a with an inhibitor of MAPKs or PI3K/Akt. Densitometric analyses of p53 (**B**) and PUMA (**C**) expression from five separate experiments. *p < 0.05 vs the control group; #p < 0.05 vs the SiO_2_ group. (**D**) MTT assay showing that SiO_2_ increased the viability of HPF-a in a time-dependent manner. *p < 0.05 vs the control group at the corresponding time point.

After confirming that MAPK and Akt activities were modulated after exposure to SiO_2_, the effect of pharmacological inhibition of these kinases was examined (Fig. 4). The purpose of these experiments was to determine whether the kinase pathways of interest (JNK, ERK, p38, and PI3K/Akt) regulate 1) the expression of p53/PUMA and 2) the extent of fibroblast migration from the nested matrix. The 30 min time point after SiO_2_ exposure was selected to maximize the probability of detecting the effects of kinase inhibition because this time point corresponded to relatively large increases in p53 and PUMA expression in HPF-a after SiO_2_ exposure (see Fig. 3A–C). Pre-treatment of HPF-a with the commercially available small molecule U0126 (20 nmol/L, MEK inhibitor), SP600125 (20 nmol/L, JNK inhibitor) or SB203580 (20 nmol/L, p38 inhibitor) abolished the SiO_2_-induced up-regulation of p53 and PUMA expression (Fig. 4A–C). However, LY-294002 (20 nmol/L, Akt inhibitor) only partially inhibited the SiO_2_-induced up-regulation of p53 and PUMA expression (Fig. 4A–C). The dosage of inhibitors was based on the manufacturers’ recommended doses and previous studies from our lab. Both efficacy and toxicity were evaluated (Supplementary Figure-S1 and Figure-S2). The results from these experiments indicated that the SiO_2_-induced expression of p53 and PUMA is mediated primarily by the MAPK pathways but not by the PI3K/Akt pathway.

### Effects of p53 and PUMA RNAi on HPF-a viability and fibroblast-populated collagen matrix (FPCM) contraction

**Figure 5:**
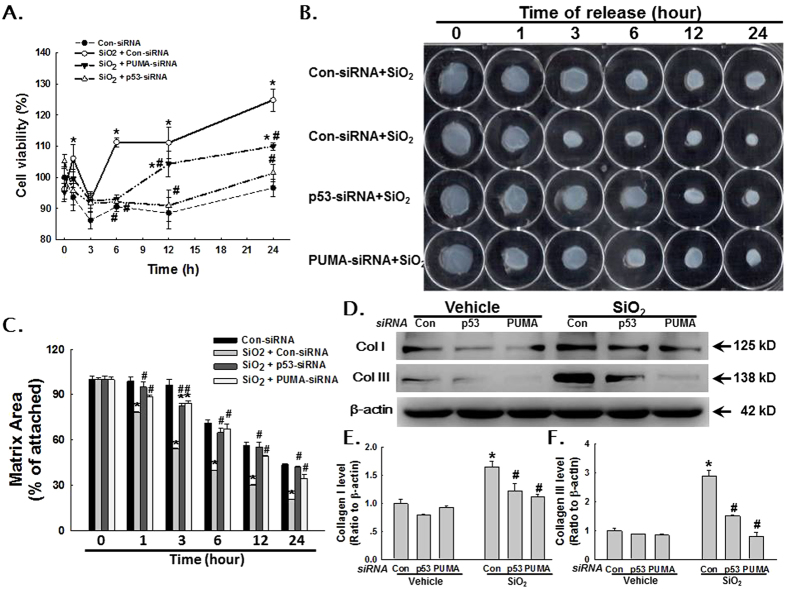
p53 and PUMA mediated SiO_2_-induced cell activation. (**A**) MTT assay showing that the SiO_2_-induced effect on cell viability was abolished by RNAi of p53 or PUMA. (n = 5) *p < 0.05 vs the control group at the corresponding time point; #p < 0.05 vs the SiO_2_ group at the corresponding time point. (**B**) Representative images of the collagen gel size showing that SiO_2_ induced increased gel contraction (indicating fibroblast activation), which was abolished by RNAi of p53 or PUMA. (**C**) Quantification of gel size at different time points after SiO_2_ exposure. (n = 6) *p < 0.05 vs the control group at the corresponding time point; #p < 0.05 vs the SiO_2_ group at the corresponding time point. (**D**) Representative Western blot showing that increased expression of collagen I and collagen III induced by SiO_2_ was attenuated by RNAi of p53 and PUMA. Densitometric analyses of collagen I (**E**) and collagen III (**F**) expression from five separate experiments. *p < 0.05 vs the vehicle group; #p < 0.05 vs the SiO_2_ group.

To understand the functional relevance of the changes in expression of p53 and PUMA, cell viability and activity were measured using the MTT assay and the collagen gel contraction assay, respectively^18^. SiO_2_ induced a significant increase in the viability of HPF-a (Fig. 4D), an effect that was abolished by RNAi of p53 and PUMA (Fig. 5A). Moreover, SiO_2_ treatment induced gel contraction in the control RNAi group, which suggested that fibroblast activity was up-regulated. However, RNAi of p53 and PUMA abolished the SiO_2_-induced increase in gel contraction (Fig. 5B,C).

### Effect of p53 and PUMA RNAi on the production of collagen from HPF-a

Previous work from our lab has shown that SiO_2_ induces rapid and sustained production of collagen from HPF-a^19^. However, whether p53 and PUMA are also involved in the production of collagen remains unknown. As shown in Fig. 5D–F, RNAi of p53 and PUMA alone had no effect on the production of collagen I or collagen III. However, RNAi of p53 and PUMA inhibited the SiO_2_-induced increase in the production of collagen I and collagen III (Fig. 5D–F).

### Effects of p53 and PUMA RNAi on the migration of HPF-a

**Figure 6:**
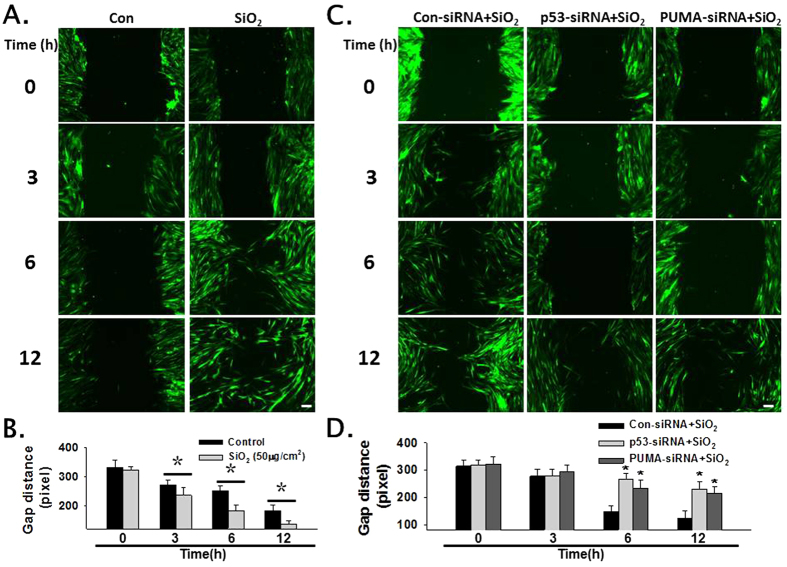
p53 and PUMA mediated HPF-a migration induced by SiO_2_ in 2D cultures. (**A**) Representative images showing that SiO_2_ induced the migration of GFP-labeled HPF-a cultured as a monolayer. Scale bar = 80 μm. (**B**) Quantification of the scratch gap distance from six separate experiments. *p < 0.05 vs the control group at the corresponding time point. (**C**) Representative images showing that SiO_2_-induced cell migration was abolished by RNAi of p53 or PUMA. Scale bar = 80 μm. (**D**) Quantification of the scratch gap distance from six separate experiments. *p < 0.05 vs the control siRNA-treated group at the corresponding time point.

Increasing evidence has suggested that pulmonary fibroblast migration is a critical aspect of pulmonary fibrosis. Therefore, we explored the roles of p53 and PUMA in SiO_2_-mediated cell migration. As shown in Fig. 6A,B, SiO_2_ induced a significant increase in the migration of HPF-a based on a scratch assay. However, RNAi of p53 and PUMA inhibited the SiO_2_-induced increase in cell migration (Fig. 6C,D).

**Figure 7:**
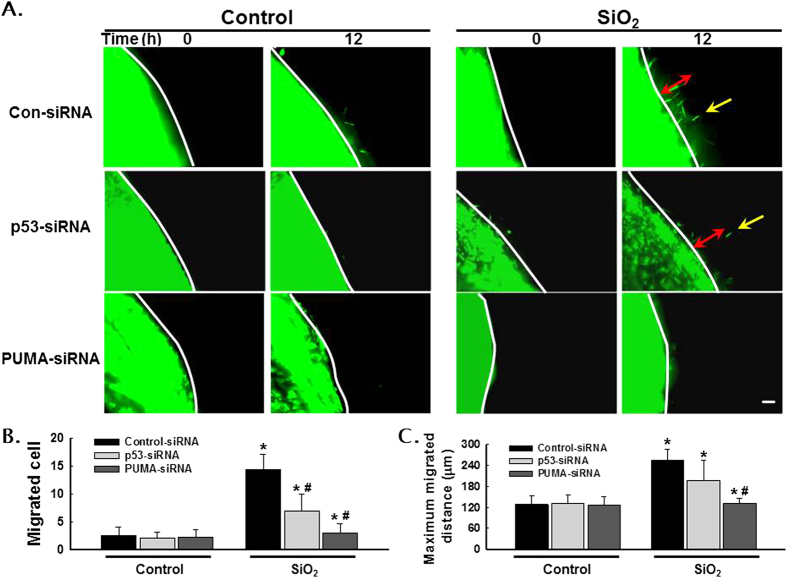
p53 and PUMA mediated SiO_2_-induced HPF-a migration in a nested matrix model. (**A**) Representative images showing that SiO_2_ induced cell migration in the nested gel matrix, which was abolished by RNAi of p53 or PUMA. Scale bar = 80 μm. Quantification of the migrated cell number from the nested gel matrix (**B**) and the maximum migrated distance (**C**) from six separate experiments. *p < 0.05 vs the corresponding control group; #p < 0.05 vs the SiO_2_- and control siRNA-treated group.

Significant differences in cell physiology between two-dimensional (2D) and three-dimensional (3D) *in vitro* culture systems have been shown^20^,^21^,^22^,^23^. An FPCM culture system has facilitated the analysis of fibroblast physiology under conditions more closely resembling the *in vivo*-like environment than do conventional 2D cell culture systems^20^,^21^,^22^,^23^,^24^. After using the scratch assay to determine that SiO_2_ exposure induces the migration of HPF-a, we sought to validate these findings by monitoring HPF-a migration in a 3D cell culture system. As shown in Fig. 7A–C, SiO_2_ induced a significant increase in cell migration in the control RNAi group, which was similar to the results of the scratch assay. Interestingly, although RNAi of PUMA abolished the increases in both the number of migrating cells and the migration distance induced by SiO_2_, RNAi of p53 only partially inhibited these parameters (Fig. 7A–C).

## Discussion

Silica exposure causes lung inflammation and fibrosis, which is histologically characterized by areas of inflammation, matrix deposition, and fibroblastic foci^25^,^26^. Various *in vitro* and *in vivo* studies have been performed to investigate the role of AMOs in SiO_2_-induced toxicity^27^,^28^,^29^,^30^. Recent studies have suggested that the direct effect of SiO_2_ on dendritic cells also plays an important role in the pathogenesis of fibrosis. In the current study, we focused on the effects of p53 and PUMA expression in pulmonary fibroblasts on cell proliferation and migration after *in vitro* exposure to SiO_2_.

MCP-1 is a well-known member of the CC subfamily of chemokines that has been linked to inflammatory disease^31^,^32^,^33^. The role of AMO-expressed MCP-1 in pulmonary fibrosis has been investigated previously^33^,^34^,^35^. Consistent with this work, our data here also showed that the MCP-1 level in both the BAL fluid and the culture medium of AMOs from silicosis patients was significantly higher than the level in samples from healthy donors (Fig. 1A,B). AMOs from healthy donors released MCP-1 in a time-dependent manner after exposure to SiO_2_ (Fig. 1E). Recent studies and the data from our lab have suggested that MCP-1 from fibroblasts is an important mediator of SiO_2_-induced fibrosis^26^,^35^,^36^. More recently, the interaction between MCP-1 and p53 has received significant attention^15^,^16^,^17^. In the current study, culture medium of AMOs from patients produced an effect on p53 expression in HPF-a similar to the effect caused by SiO_2_ (Fig. 1C,D). These results indicated that p53 may mediate the effect of MCP-1 on SiO_2_-induced fibrosis.

Numerous studies have explored the role of p53 in cell activation and migration. For example, increased p53 expression was related to a decrease in fibroblast migration in the presence of firsthand cigarette smoke in a 2D *in vitro* model^37^. Accumulating evidence has suggested a functional association between p53 and the regulation of cell morphology and motility. For example, a recent study suggested that p53 may play a role in Cdc42-mediated filopodia formation and cell polarization^38^. Moreover, the p53 tumor suppressor pathway is functionally connected to the Rho GTPase pathways in regulating cell-ECM and cell-cell adhesions, as well as cell invasion properties^39^. However, the p53-regulated genes that are required for SiO_2_-induced fibrosis remain unclear. PUMA is well known for its role in mediating apoptotic responses induced by p53. For instance, PUMA deficiency in mice decreased mouse embryonic fibroblast apoptosis^40^. The paradoxical finding that PUMA promotes angiogenesis by increasing cell proliferation and survival has also been reported^14^. In fact, little is known regarding the function of PUMA in pulmonary fibroblasts. In this study, we found that both p53 and PUMA expression increased after exposure to SiO_2_. Consistent with this, fibroblast activity also increased, as indicated by the elevated expression of α-SMA and by the results of the gel contraction assay. Therefore, the MAPK and PI3K pathways are involved in the SiO_2_-induced up-regulation of p53 and PUMA expression, which was abolished by pretreatment with an inhibitor of MAPKs or PI3K. Together, these findings have helped elucidate the molecular mechanism underlying the regulation of p53 and PUMA.

In this study, we also investigated the effects of p53 and PUMA up-regulation on cell proliferation and migration. Increasing evidence has suggested that fibroblast proliferation and migration increase during silicosis. In our *in vitro* model of silicosis, the direct effects of SiO_2_ on HPF-a were consistent with previous findings. Both p53 and PUMA silencing via RNAi treatment attenuated HPF-a proliferation and activation induced by SiO_2_, as indicated by the MTT and gel contraction assays. Interestingly, RNAi of PUMA only partially abolished SiO_2_-induced cell proliferation, which indicated that the effect of p53 on fibroblasts may be mediated by both PUMA-dependent and -independent pathways. In fact, p53 was recently reported to exert its apoptotic effects via Noxa and Bim^40^. Whether Noxa and Bim are also involved in p53-mediated activities in silicosis requires further investigation. Nevertheless, the proliferative effect of both p53 and PUMA was surprising because this finding suggested an important therapeutic strategy for silicosis.

To investigate SiO_2_-induced cell migration, we applied both the convenient scratch assay and the nested matrix migration assay. Due to discrepancies in cellular behavior between 2D and 3D culture systems, the FPCM *in vitro* culture model provides opportunities to investigate cell-cell and cell-matrix interactions in an environment that more closely resembles physiological conditions^41^. Cells in 3D matrices display distinct patterns of morphology and migration from those on a 2D monolayer. For instance, when fibroblasts are cultured on a 2D monolayer surface, the fractional force against the stiff substrate results in cell migration^42^,^43^. However, when these cells are cultured in 3D collagen matrices, the fractional force can be utilized as a mechanical inducer of matrix remodeling both locally and globally. Therefore, understanding the molecular mechanism underlying fibroblast migration in a 3D matrix may be important for understanding various pathological diseases such a fibrosis^23^. In this study, the results from the 2D scratch assay revealed that RNAi of p53 and PUMA abolished the increases in cell migration induced by SiO_2_. However, in the 3D nested matrix migration model, while RNAi of PUMA abolished the cell migration induced by SiO_2_, RNAi of p53 only partially abolished this effect. One explanation for this result is that p53 exerts its effect via a PUMA-independent pathway. Alternatively, PUMA may induce apoptosis in a p53-independent manner in response to a wide variety of stimuli, including genotoxic stress, deregulated oncogene expression, toxins, altered redox status, growth factor/cytokine withdrawal and infection^44^. Recently, the role of PUMA in fibroblast activation has received significant attention. For instance, PUMA deletion attenuated pressure overload–induced apoptosis and fibrosis in mice subjected to heart failure^45^,^46^. One interesting finding is that RNAi of p53 only attenuated the increase in the number of migrated cells but not in the migrated distance, which indicated a complex interaction between p53 and PUMA.

The limitation of our current study was that the fibroblasts used were purchased from a company that obtained the cells from one donor. Despite this single source, the fibroblasts have displayed phenotypic and functional heterogeneity, even when isolated from the same tissue^47^,^48^,^49^,^50^,^51^. Importantly, however, previous work has shown that the growth rate pattern of the heterogenetic subpopulation obtained from one donor closely resembles those of subpopulations generated from other donors, indicating that heterogeneity is a consistent characteristic of fibroblasts^51^. Nevertheless, experiments using different donors will worth conducting in future investigations.

In summary, our findings demonstrated that SiO_2_ induces the expression of p53 and PUMA. Both p53 and PUMA play a vital role in pulmonary fibroblast activation and migration. The regulation of p53 and PUMA expression and function has potential as a novel therapeutic strategy for the treatment of silicosis.

## Materials and Methods

### Reagents

Fetal bovine serum (FBS), normal goat serum (NGS), Dulbecco’s modified Eagle’s medium (DMEM; #1200-046), and 10X MEM (11430-030) were purchased from Life Technologies^TM^. Amphotericin B (BP2645) and GlutaMax^TM^ Supplement (35050-061) were obtained from Gibco®, and Pen Strep (15140-122) was obtained from Fisher Scientific. PureCol® type I bovine collagen (3 mg/mL) was obtained from Advanced Biomatrix. Antibodies against p53 (SC6243), PUMA (SC374223) and β-actin (SC8432) were obtained from Santa Cruz Biotechnology®, Inc. The antibody against α-SMA (SAB5500002) was purchased from Sigma, Inc.

### Isolation of alveolar macrophages from human lung bronchoalveolar lavage fluid (BALF)

The use of primary AMOs derived from human lung BALF was approved in accordance with the approved guidelines of the Research and Development Committee of the First Affiliated Hospital of Nanjing Medical University. After obtaining informed consent from the subject, AMOs were isolated from bronchoalveolar lavage. This procedure was performed using a flexible fiber optic bronchoscope under local anesthesia of the upper airway with 2% lidocaine, as described previously^52^,^53^. Briefly, the bronchoscope was wedged into the subsegmental bronchus of the right middle lobe or, in patients with EP, into areas of lung parenchyma that were otherwise normal based on chest roentgenography. Then, 150 mL of normal saline was instilled in 50-mL aliquots. Harvested BALFs were filtered through sterile nylon mesh and centrifuged at 160 × g for 10 min to obtain the cell preparation.

Macrophages were separated from the cell preparation by differential centrifugation using a Percoll solution. Macrophages isolated by this procedure exceed 95% purity^53^. The isolated cells were plated in a sterile T-25 flask at 37 °C and equilibrated with 5% CO_2_ until the macrophages firmly adhered to the flask. Cell viability was assessed according to Trypan blue exclusion.

### HPF-a culture

HPF-a were purchased from ScienCell. The cells were maintained in T75 flasks in DMEM containing 10% FBS. HPF-a were stored at passages 3–7 (P3-7) in liquid nitrogen. A vial of P3-7 HPF-a between P10 and P15 was thawed, seeded, and passaged upon confluence to perform each experiment. We did not observe any correlation between passage number and any of the investigated parameters using cells in this range.

### Lentiviral transduction of primary HPF-a

HPF-a were transduced with LV-RFP lentivirus (Hanbio, Inc., Shanghai, CN) as described previously^41^. Briefly, P3-4 primary HPF-a were cultured in a 24-well plate (1 × 10^4^ cells/well) in DMEM containing 10% FBS for 48 h. Then, the medium was replaced with 1 mL of fresh medium containing 8 μg/mL polybrene. Next, 100 μL of lentivirus solution (10^7^ IU/mL) was added to each well, followed by incubation at 37 °C and 5% CO_2_ for 24 h. After incubation, the treatment medium was replaced with fresh DMEM containing 10% FBS, and the cells were cultured at 37 °C and 5% CO_2_ until the cells reached >50% confluence. The transduced cells were selected using blasticidin as follows. Briefly, the medium was replaced with DMEM containing 10 μg/mL puromycin and 10% FBS, and the cells were cultured at 37 °C and 5% CO_2_ for 24 h. Then, the cells were washed twice with fresh DMEM containing 10% FBS. Pure transduced HPF-a cultures were expanded and/or stored in liquid nitrogen as described previously^54^.

### MTT assay

Cell viability was measured via the 3-(4,5-dmethylthiazol-2-yl)-2,5-diphenyl tetrazolium bromide (MTT) method. Briefly, the cells were collected and seeded in 96-well plates. Different seeding densities were employed at the beginning of the experiments. The cells were then exposed to I/R medium. Following incubation for different periods (3–24 h), 20 μl of MTT dissolved in Hank’s balanced salt solution was added to each well at a final concentration of 5 μg/ml, and the plates were incubated in a 5% CO_2_ incubator for 1–4 h. Finally, the medium was aspirated from each well, and 200 μl of dimethyl sulfoxide was added to dissolve the formazan crystals. Then, the absorbance of each well was obtained using a plate reader at reference wavelengths of 570 nm and 630 nm. Each of the experiments was repeated at least three times.

### Gel contraction assay

Fibroblast-populated collagen matrix (FPCM) contraction was determined using the floating matrix contraction assay as described previously^55^ with minor modifications. Briefly, the matrices were polymerized, covered with DMEM containing 5% FBS, released from the culture well using a sterile spatula, and incubated at 37 °C. At different time points after the matrices were released, they were fixed in 4% paraformaldehyde in phosphate-buffered saline (PBS) at 4 °C overnight, and images were obtained using a desktop flatbed scanner. The matrix area was measured using ImageJ software, and the data are presented as the ratio of the released matrix area to the attached matrix area.

### *In vitro* scratch assay

Cell migration ability in the 2D culture system was evaluated using an *in vitro* scratch assay. Briefly, 1 × 10^5^ HPF-a were seeded in 24-well tissue culture plates and cultured in growth medium for 24 h, at which time they were approximately 70–80% confluent. Using a sterile 200-μL pipette tip, a straight line was carefully scratched in the monolayer across the center of the well in a single direction while maintaining the tip perpendicular to the plate bottom. A second straight line was similarly scratched perpendicular to the first line to create a cross-shaped cellular gap in each well. Each well was then washed twice with 1 mL of fresh growth medium to remove any detached cells. Digital images of the cell gap were captured at different time points, and the gap width was quantitatively evaluated using ImageJ software.

### FPCM

The collagen matrix model was utilized as described previously^54^,^56^. The final matrix parameters were as follows: volume = 0.2 mL; diameter = 12 mm; collagen concentration = 1.5 mg/mL; and cell concentration = 1.0 × 10^6^ cells/mL. The matrices were established in 24-well plates (BD#353047), and the cells were incubated in the attached state in DMEM containing 5% FBS for approximately 48 h before the initiation of each experiment.

### Nested matrix model and cell migration assay

The nested collagen matrix model was utilized as described previously^57^ with some modifications. For the nested attached matrix, a standard FPCM was incubated in the attached state for 72 h in DMEM containing 10% FBS. Then, the FPCM was removed from the culture well and placed in a 60-μL aliquot of fresh acellular collagen matrix solution (a NeoMatrix solution) that was centered inside a 12-mm diameter score on the bottom of a new culture well. Next, a 140-μL aliquot of NeoMatrix solution was used to cover the newly transferred FPCM. The NeoMatrix was allowed to polymerize for 1 h at 37 °C and 5% CO_2_, and then 2 mL of DMEM containing 10% FBS was added to the well.

Cell migration from the nested FPCM to the acellular NeoMatrix was quantified via fluorescent microscopy 24 h after nesting. Digital images (constant dimensions of 1000 × 800 μm) were captured using an EVOS® FL Cell Imaging microscope (Life Technologies, Grand Island, NY, USA) from 3–5 randomly selected microscopic fields at the interface between the nested FPCM and the acellular NeoMatrix. HPF-a migration from the nested FPCM was quantified by counting the number of cells that had clearly migrated from the nested matrix to the cell-free matrix. The maximum migration distance was quantified by identifying the cell that had migrated the longest distance from the nested matrix to the cell-free matrix. The number of cells per field that had migrated from the nested matrix and the maximum migration distance per field was averaged from these digital micrographs.

### Immunoblotting

Immunoblotting was utilized as described previously^54^ with minor modifications. HPF-a were collected from the culture dishes, washed with PBS and lysed using a mammalian cell lysis kit (MCL1-1KT, Sigma-Aldrich®) according to the manufacturer’s instructions. The Western blot membranes were probed with primary antibodies. Alkaline phosphatase-conjugated goat anti-mouse or anti-rabbit IgG secondary antibodies were used (1:5,000). The signals were detected using chemiluminescence (SuperSignal West Dura Chemiluminescent Substrate, Thermo Scientific). Each Western blot analysis was repeated using cells from three different donors. A single representative immunoblot is shown in each figure. Densitometry was performed using ImageJ software, and the results for all the repeated experiments were combined into one plot.

### Immunocytochemistry

HPF-a were fixed in 4% paraformaldehyde in PBS at 4 °C overnight. Then, the fixed samples were permeabilized for 30 min at room temperature (RT) with 0.3% Triton X-100 in PBS. The permeabilized samples were blocked with PBS containing 10% NGS (Life Technologies) and 0.3% Triton X-100 at RT for 2 h. The blocked samples were incubated in primary antibodies in PBS containing 10% NGS and 0.3% Triton X-100 at 4 °C overnight. Then, the samples were washed three times with PBS and incubated in donkey anti-rabbit (conjugated to Alexa-Fluor® 488) and donkey anti-mouse (conjugated to Alexa-Fluor® 576) secondary antibodies for 2 h at RT. After the samples were washed three times in PBS, they were mounted using Prolong® Gold antifade reagent with DAPI (P36931, Life Technologies). The slides were examined under an EVOS FL fluorescence microscope.

### RNA interference of p53 and PUMA using siRNA

RNA interference targeting p53 and PUMA was performed on HPF-a as described previously^58^ with some modifications. The RNA interference protocol for a single well of a 24-well plate was as follows. Briefly, 49 μL of serum-free DMEM was combined with 1 μL of transfection reagent, and 1 μL of siRNA stock was added to 49 μL of serum-free DMEM. Then, both solutions were incubated at RT for 15 min. The transfection reagent and siRNA solutions were mixed together, and the resulting solution was incubated at RT for an additional 15 min. Meanwhile, HPF-a were seeded at a concentration of 5.0 × 10^5^ cells/100 μL/well in serum-free DMEM. The siRNA-vehicle solution was mixed and incubated at RT for 15 min. The siRNA-vehicle solution was added to the plated cells. The transfected HPF-a were cultured in serum-free DMEM for 24 h. Then, the medium was replaced with DMEM containing 10% FBS for 48 h before conducting further experiments. The siRNA knockdown efficiency was determined 2 days after transfection via Western blot analysis.

### Statistics

The data are presented as the means ± SEM. Unpaired numerical data were compared using an unpaired t-test (two groups) or ANOVA (more than two groups), and statistical significance was set at p < 0.05.

## Additional Information

**How to cite this article**: Wang, W. *et al.* p53/PUMA expression in human pulmonary fibroblasts mediates cell activation and migration in silicosis. *Sci. Rep.* **5**, 16900; doi: 10.1038/srep16900 (2015).

## Supplementary Material

Supplementary Figure
