# Supplementary material for: p53/PUMA expression in human pulmonary fibroblasts mediates cell activation and migration in silicosis
Source: Sci Rep. 2015 Nov 18;5:16900. doi: 10.1038/srep16900 (PMC4649630; doi:10.1038/srep16900)
Supplement: Supplementary Figure [file srep16900-s1.pdf]

Supplementary Information

Manuscript title: p53/PUMA expression in human pulmonary fibroblasts mediates cell activation and migration in silicosis

Authors: Wang, Liu, Dai, Fang, Wang, Zhang, Yao, Zhang, Chao

Table of Contents

Supplementary Figure S1 2

Supplementary Figure S2 3

Supplementary Figure-S1

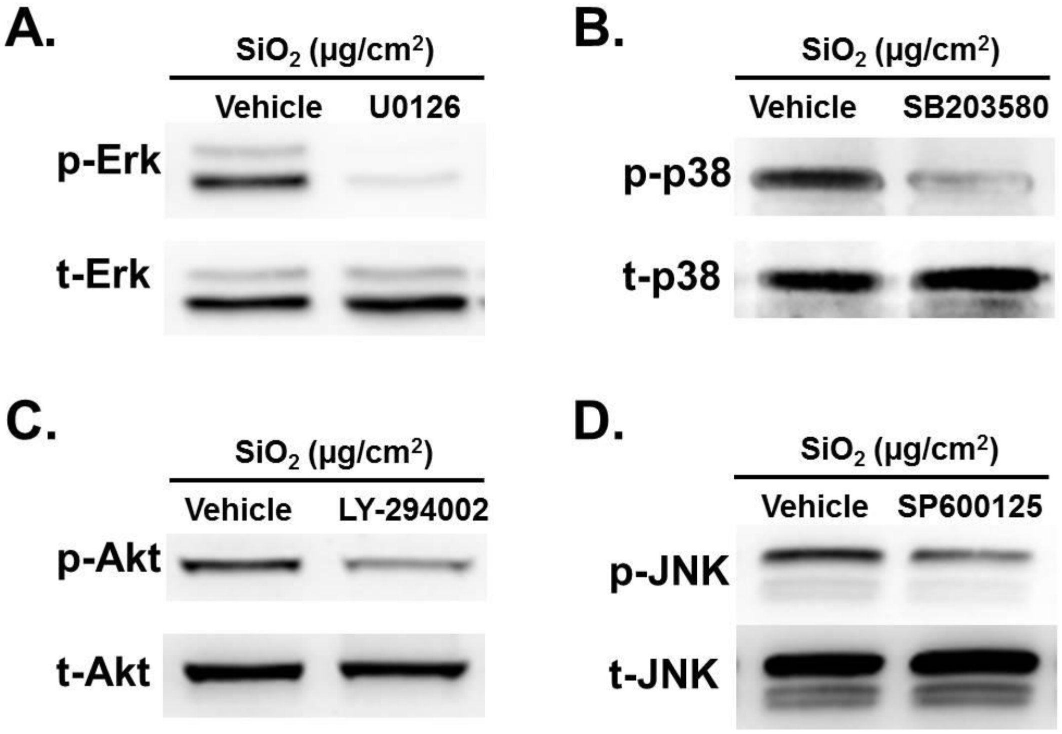

Sup-Figure-S1

Representative Western blot showing that phosphorylation of MAPKs or PI3K/Akt induced by SiO_2 was abolished by pretreating HPF-a with a corresponding inhibitor of MAPKs or PI3K/Akt. (n=3)

Supplementary Figure-S2

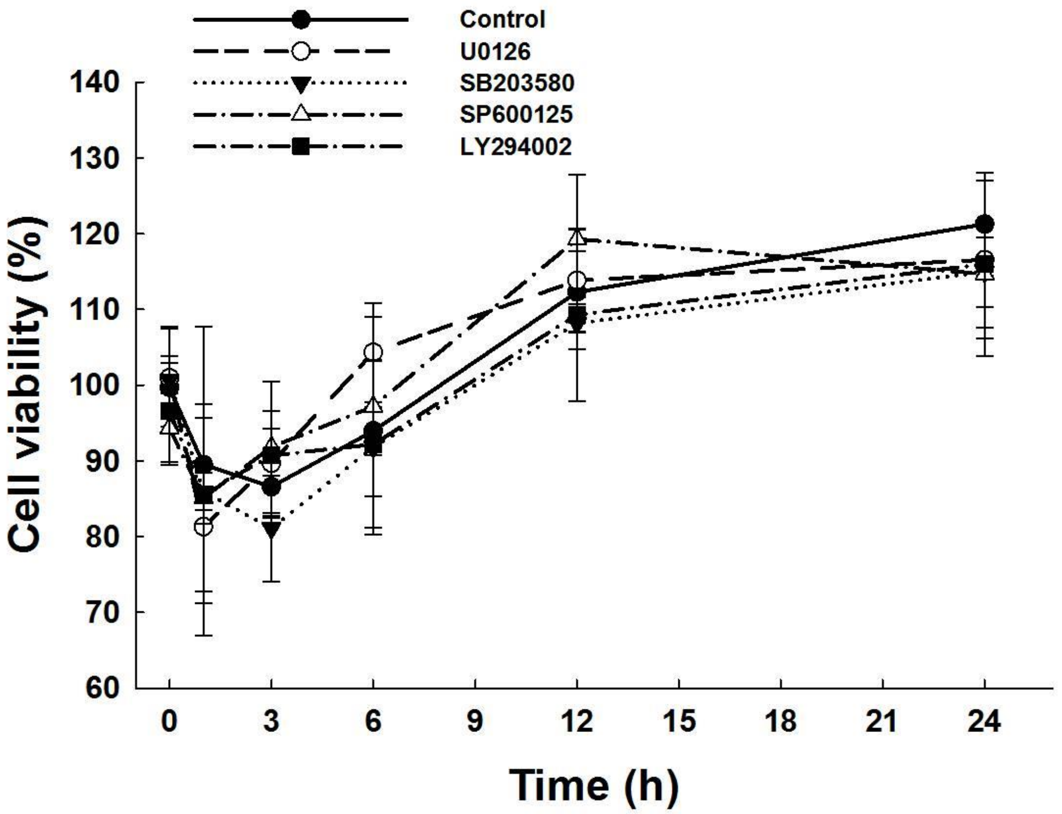

Sup-Figure-S2

MTT assay showing that inhibitor of MAPKs or PI3K/Akt had no effect on cell viability of HPF-a. (n=5)
